# Should Systematic HSV Serological Screening of Donors Be Implemented to Manage Mismatched HSV D+/R− Liver Transplants?

**DOI:** 10.3389/ti.2025.14835

**Published:** 2025-07-21

**Authors:** J. Besombes, V. Thibault, A. Bacle, C. Bressollette-Bodin, C. Camus, B. Giguet, P. Houssel-Debry, C. Pronier

**Affiliations:** ^1^ Virology Department, Rennes University Hospital, Rennes, France; ^2^ Univ Rennes, Inserm, EHESP, Irset (Institut de recherche en santé, environnement et travail), UMR_S 1085, Rennes, France; ^3^ Pôle Pharmacie, Service Hospitalo-Universitaire de Pharmacie, Centre hospitalo-universitaire de Rennes, Rennes, France; ^4^ Nantes Université, Centre Hospitalo-universitaire de Nantes, Service de virologie, Nantes, France; ^5^ Nantes Université, Center for Research in Transplantation and Translational Immunology, UMR 1064, Nantes, France; ^6^ Rennes University Hospital, Intensive care unit, Rennes, France; ^7^ Liver Disease Department, Rennes University Hospital, Rennes, France

**Keywords:** liver transplant, donor screening, herpes simplex virus, prophylaxis, Immunosuppression

Dear Editors,

Solid organ transplant (SOT) recipients are at high risk of infection due to immunosuppressive therapy, particularly in the early post-transplant period. Although guidelines for cytomegalovirus (CMV) are well-established, herpes simplex virus (HSV) recommendations are less clear and vary among countries. CMV risk management strategy for SOT recipients depends on the serological status of the donor (D) and the recipient (R). In liver transplantation, mismatched CMV-D+/R- or R+ patients may receive valganciclovir prophylaxis which is typically shorter for R+. Alternatively, they may undergo a preemptive approach based on regular CMV PCR monitoring [1–4]. Current guidelines HSV-specific prophylaxis (acyclovir or valacyclovir) primarily target HSV-R+ patients without prophylaxis for CMV and some of them propose to also consider HSV-D+/R- patients (Supplementary Material S1) [1–3, 5–8]. Transmission may occur through close contact or be donor-derived and can lead to moderate or severe infections, particularly in immunocompromised patients. However, while screening for several viral infections is routinely performed in donors, HSV serology is not consistently included—often due to the presumed high seroprevalence. This letter aims to highlight this paradox, which hinders the optimal management of patients following the transplantation. To illustrate, we describe our 2023 liver transplant (LT) cohort—from the second largest center nationally—based on CMV and HSV serological status and discuss the management of two mismatched HSV-D+/R- cases.

In 2023, our center performed 122 LT, with recipients averaging 55.3 ± 12.1 years of age, the majority of whom were men (83/122; 68.0%). HSV1/2 IgG testing (Liaison^®^ XL HSV-1/2 IgG, DiaSorin), is included in recipient screening prior to transplantation, without distinction between anti-HSV-1 and HSV-2 IgG. Among the 122 LT recipients tested, 77.0% (94/122) were positive, 19.7% (24/122) negative, and 3.3% were uncertain (4/122) as signals were near the assay’s detection threshold (Table 1A). Regarding CMV IgG status, 39.3% (48/122) of patients were positive and 60.7% (74/122) negative (Table 1A). HSV-R+ patients benefit from a clinical monitoring regardless of CMV prophylaxis. Among HSV-seronegative or uncertain status recipients, 21.4% (6/28) were CMV-D±/R+, 39.3% (11/28) CMV-D-/R- and 39.3% (11/28) CMV-D+/R- (Table 1B). Mismatched CMV-D+/R-patients received valganciclovir prophylaxis (450-mg twice daily if normal renal function) for 3 months, which may be effective in preventing HSV infection. No specific HSV virological follow-up was planned. Patients with CMV-R+ status benefit from a preemptive approach and CMV-D-/R-patients only benefit from a clinical follow-up. In all cases, there is no HSV systematic monitoring. Notably, 60.7% (17/28) of HSV-R- did not receive any antiviral prophylaxis or specific follow-up to prevent a possible primary HSV infection that could eventually be transmitted by the donor or close contact (Table 1B). In our cohort, knowledge of the HSV donor status could have been relevant for at least 14% (17/122) of the recipients but clinicians requested HSV serology for only two cases. In the first case, a 67-year-old male recipient was HSV seronegative, prompting the clinician to request HSV serology. The donor was HSV seropositive. The patient ultimately received valganciclovir prophylaxis due to a CMV D+/R–status. In contrast, the second case, a 43-year-old female did not receive any prophylaxis because she was CMV-D-/R- (Supplementary Material S2). She developed primary HSV-1 infection 27 days post-transplantation (DPT) and presented with acute hepatitis and gingivostomatitis. Retrospective HSV nucleic acid test (NAT) revealed that active HSV infection began very early (6 DPT). After two negative plasma HSV NAT results during acyclovir treatment and clinical improvement, a secondary prophylaxis with valacyclovir was continued for 1 month. After the diagnosis of HSV infection in the recipient, retrospective testing of the donor confirmed HSV-seropositivity. HSV DNA was also detected in the donor’s respiratory and blood (<107 copies/mL; ct = 35; HSV-1 HSV-2 R-GENE^®^) samples, potentially contributing to early transmission and infection in the recipient. This case highlights the potential benefit of systematic HSV donor screening to guide early post-transplant management.

**TABLE1:** Serological status for Herpes Simplex Virus (HSV) and Cytomegalovirus (CMV) in patients undergoing liver transplantation in the year 2023 in a French center. Data extracted from laboratory information system. Serologies performed on the Liaison XL, DiaSorin®.

A	anti-HSV 1/2 IgG		anti-CMV IgG	
	n	%	n	%
Total	**122**	**-**	**122**	**-**
Positive	94	77.0	48	39.3
Negative	24	19.7	74	60.7
Uncertain	4	3.3	0	0

B	HSV negative or uncertain serostatus (n = 28)			
	n	%	Recipient management	Protection against HSV
CMV R+	6	**21.4**	CMV virological monitoring with pre-emptive treatment	No
CMV D-/R-	11	**39.3**	No special follow-up (CMV PCR if clinical signs)	No
CMV D+/R-	11	**39.3**	**Prophylaxis by valganciclovir during 3 months**	**Yes**

A. Anti-HSV IgG (with no distinction between HSV-1 and 2) and anti-CMV IgG status of the 122 liver transplants patients. B. Anti-CMV IgG donor-recipient status in HSV-seronegative patients and management recipient in the centre. R, recipient; D, donor; uncertain, ratio around the technique’s detection threshold.

Guidelines for HSV prophylaxis in SOT recipients generally recommend prophylaxis for HSV-R+ based on CMV status. Although prophylaxis reduces the risk of HSV reactivation, the lack of data on HSV-related morbidity in HSV-R+ limits our ability to fully assess the disease burden underlying this recommendation [9]. American Society of Transplantation recommends HSV-specific prophylaxis for at least 1 month in HSV-R+ without CMV prophylaxis and suggest it only at the clinician’s discretion for R-patients, without specifying a duration [1]. Recent European guidelines recommend HSV prophylaxis (e.g., valacyclovir) for D+/R− recipients without CMV prophylaxis. If the donor’s status is unknown and the recipient is seronegative, management should follow the D+/R− approach [3]. This is consistent with the proposals by Arana et al. in 2022 [5]. Although HSV donor serology may be useful for diagnostic purposes, there are currently no guidelines recommending universal HSV screening in donors. Only the Swiss Transplant Infectious Diseases working group modified its national recommendations and proposed pretransplant HSV serostatus determination in liver recipients and donors to guide HSV prophylaxis in HSV-D+/R- mismatches [6]. Indeed, HSV serologic testing in donors is generally not recommended based on the known globally high HSV seroprevalence. Nonetheless in Northern countries, fewer than 60% and 20% of people under the age of 50 are infected with HSV-1 and HSV-2, respectively [10–12]. Besides, HSV-1/2 seroprevalence is decreasing by 1% per year, particularly among young people in Europe and the United States [1, 10, 11]. In 2023, about 1 in 5 LT recipients were HSV-seronegative in our center, a proportion expected to rise. As donor age increases, young organ recipients may face a higher risk of contracting HSV from HSV-D+. Despite the constantly evolving landscape of infectious disease screening, the lack of recommendations for HSV screening in donors highlights inconsistencies in HSV prevention.

HSV infection in SOT recipients should not be overlooked, especially given the decreasing HSV seroprevalence. Based on our experience and in line with the recent recommendations from the European council and Switzerland, we advocate routine HSV serological testing of donors to guide early post-transplant management, especially in HSV-D+/R-mismatched patients. In absence of CMV prophylaxis (Valganciclovir), HSV-specific prophylaxis (e.g., Valacyclovir) should be initiated for at least 1 month (Supplementary Material S3). Upon detection of CMV DNAemia during follow-up, valacyclovir can be stopped when valganciclovir is initiated. While universal prophylaxis for seronegative recipients without known donor status is recommended in some centers, including in European and Australian guidelines, we prefer a more individualized approach to limit patient exposure to multiple pharmacological agents [2]. Further studies are needed to determine whether a systematic approach to HSV donor screening, universal prophylaxis or a preemptive approach (weekly HSV PCR monitoring, similar to CMV protocols), would be the most cost-effective and clinically appropriate strategy for early post-transplant care. Given HSV’s tropism for certain grafts—particularly the liver—and the potential severity of primary infection, prevention should remain a priority. This is especially important as CMV prophylaxis duration may be shortened in the future based on CMV-specific cell-mediated immunity (CMV-CMI) results, which must not distract from the risk of HSV infection. Since novel CMV antivirals like letermovir—currently recommended for kidney transplant prophylaxis and expected to be approved for other organs—do not cover HSV, ensuring adequate HSV-specific prophylaxis remains essential to prevent HSV infection [4]. Lastly, behavioral counseling should be provided to reduce the risk of transmission in all HSV-seronegative recipients.

## Data Availability

The raw data supporting the conclusions of this article will be made available by the authors, without undue reservation.
