# Supplementary material for: Should Systematic HSV Serological Screening of Donors Be Implemented to Manage Mismatched HSV D+/R− Liver Transplants?
Source: Transpl Int. 2025 Jul 21;38:14835. doi: 10.3389/ti.2025.14835 (PMC12318879; doi:10.3389/ti.2025.14835)
Supplement: Supplementary file 1 [file DataSheet1.pdf]

Supplementary file 1: ***Summary of recommendations for management of the risk of herpes simplex virus (HSV) infection after transplantation.***

*SOTR = Solid Organ Transplant Recipient; D = Donor, R = Recipient, IS= immunosuppression; LT = Liver transplantation *Except in centers proposing universal antiviral prophylaxis; @ No comment about letermovir CMV prophylaxis; # depending on the intensity of the immunosuppression*

Guidelines (year)	Region	Universal pre-transplant HSV serological screening		HSV specific antiviral prophylaxis		
		[donor]	[recipient]	recommended for	with (agents commonly used)	for (duration)
European Committee on Organ Transplantation. Guide to the quality and safety of organs for transplantation (**2025**)	Europe	Not recommended	Yes	**All HSV-D+/R- or HSV-R- with unknown donor HSV serostatus without CMV antiviral prophylaxis adequate for HSV prevention**	**Valacyclovir**	Not specified
Swiss national recommendations (**2023**)	Europe	For all liver donors	Yes	**Liver recipients with a CMV preemptive follow-up strategy and an unknown HSV serology**	**Valacyclovir** starting 24 h following LT	**3 – 6 months** in case of an HSV D+/R− constellation #
Arana and colleagues from Hospital Clínic Barcelona - Spain (**2022**)	Europe	Not recommended	Yes	**HSV seronegative SOTR** without CMV antiviral prophylaxis	**Acyclovir** (eg, 400-800 mg twice a day), **valacyclovir** (500 mg twice a day), or **famciclovir** (500 mg twice a day)	During the **first month**
Spanish consensus (**2020**)	Europe	Not recommended	Unspecified	No recommendation		
The Transplantation Society of Australia and New Zealand (**2023**)	Australia	Not recommended	No	**All SOTR without CMV antiviral prophylaxis@**	Acyclovir, famciclovir, or valaciclovir	**≥ 1 month**
South Asian Transplant Infectious Disease (**2023**)	Asia	No recommendation		No recommendation		
American Society of Transplantation Infectious Diseases Community of Practice (**2019**)	America	Not recommended	Yes*	HSV-seropositive SOTR without CMV antiviral prophylaxis adequate for HSV prevention (e.g. letermovir …)	**Acyclovir** (eg, 400-800 mg twice a day), **valacyclovir** (500 mg twice a day), **or famciclovir** (500 mg twice a day)	**≥ 1 month** & during treatment of rejection episodes
				**HSV-seronegative SOTR without CMV antiviral prophylaxis: decision up to the clinician**		Not specified

<u>Supplementary file 2:</u> ***Clinical and biological monitoring of patient 2***. *Anti-HSV-1/2 IgG and HSV DNA detection were performed using the Liaison® XL HSV-1/2 IgG kit (DiaSorin) and the RealStar® HSV PCR Kit 1.0 (Altona), respectively. ALT = Alanine Aminotransferase ; ct = cycle threshold ; IV = Intravenous PRBCs = Packed red blood cells ; MMF = Mycophenolate mofetil ; N=Normal ; NT = Not tested ; Uncertain = above threshold limit. NB :HSV DNA not detected in liver biospy on D0. *Retrospective analysis of recipient plasmas collected for CMV monitoring following a positive result on D+27.*

Day prior (D-X) or post transplantation (D+X)	Clinical events	ALT	HSV DNA (Plasma)	Anti-HSV-1/2 IgG	Immunosuppressive therapy	Antiviral therapy
D-77	Registration on the transplant waiting list	N	*NT*	Negative		
D-61	PRBCs transfusion	N	*NT*	Positive		
D0	Liver transplantation	N	Not detected*	Negative	- Tacrolimus<br>- MMF<br>- Corticosteroid	
D+6	None	5*N	Detected*<br>(3.6 log UI/mL ; 35 ct)	*NT*		
D+16	- Intermittent fever<br>- Persistent pain<br>- Diarrhea	N	Detected*<br>(not quantified ; ct 28)	*NT*	- Stop Tacrolimus (overdose)<br>- MMF<br>- Corticosteroid	
D+27	Fever without localizing signs	4*N	Detected<br>(8.7 log IU/mL ; ct 19)	Uncertain	- Restart Tacrolimus<br>- Stop MMF<br>- Corticosteroid	Introdution Acyclovir (10mg/kg/8h)
D+29	Gingivostomatis	2*N	*NT*	Positive		Switched to *per os* Valacyclovir (two 500-mg tablets twice daily)
D+37	Normalisation liver function	N	Detected<br>(4.6 log IU/mL ; ct 32)	*NT*	- Tacrolimus<br>- Oral corticosteroid reduction	
D+44	Improvement	N	Detected<br>(3.9 log IU/mL ; ct 34)	*NT*		
D+56	Regular follow-up	N	Not detected	*NT*	- Tacrolimus<br>- MMF<br>- Oral corticosteroid dose reduction	
D+87	Regular follow-up	3*N	Not detected	*NT*		Switched to prophylactic Valacyclovir (one 500-mg tablet, twice daily) for 1 month

Supplementary file 3: ***Proposed Algorithm for Managing Herpes Simplex Virus Type 1 and 2 (HSV-1/2) Infection Risk in Liver Transplant Recipients (R) and Deceased Donors (D) based on Cytomegalovirus (CMV) status.*** *Patients CMV D+/R-, D±/R+ and CMV D-/R- benefit from different strategies: an antiviral prophylaxis, a preemptive therapy or a simple clinical surveillance, respectively. The presence of IgG is used to determine prior exposure of both donors and recipients to CMV and HSV infections.*

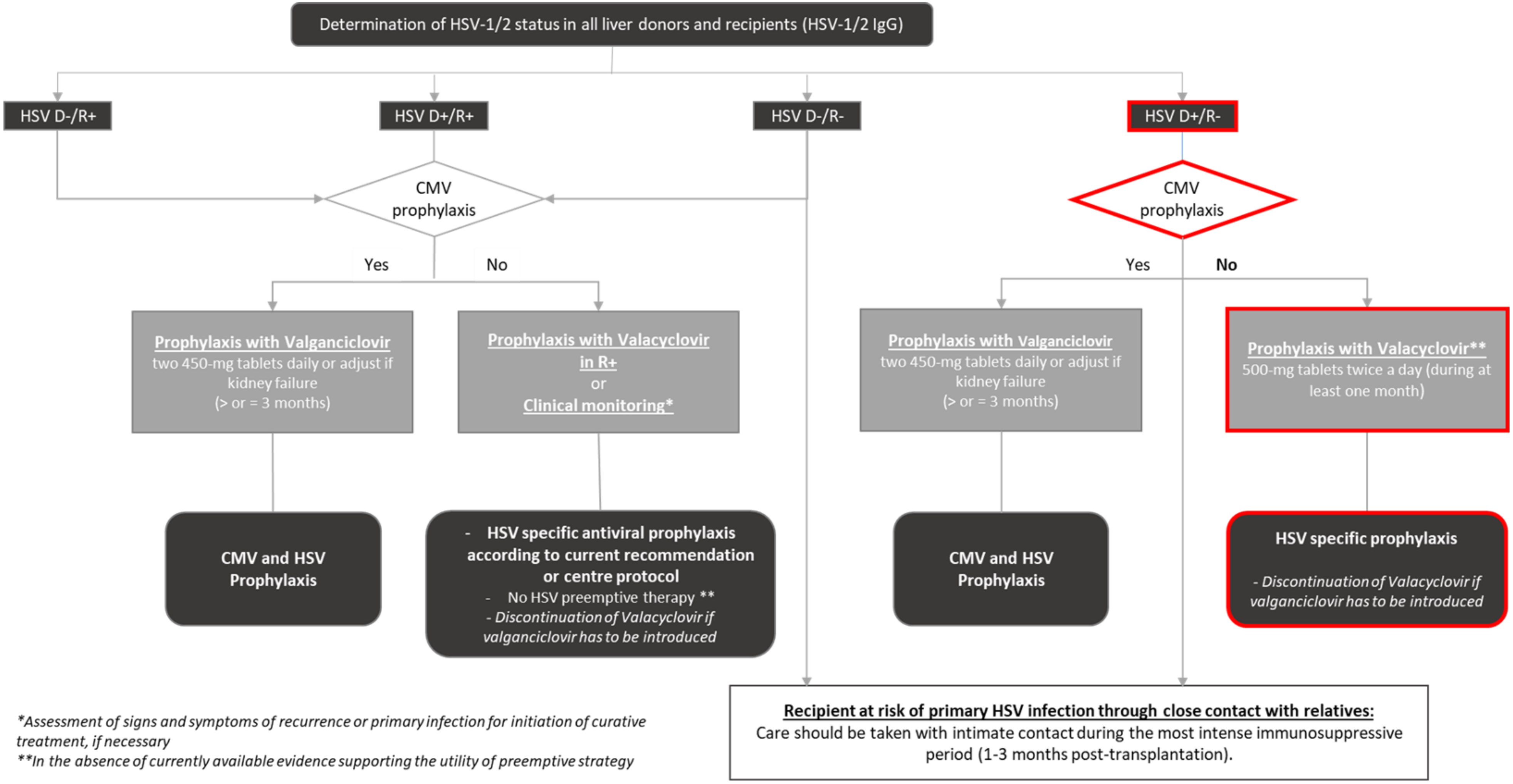

**Assessment of signs and symptoms of recurrence or primary infection for initiation of curative treatment, if necessary*
***In the absence of currently available evidence supporting the utility of preemptive strategy*
